# Psychosis in a 15-Year-Old Female with Herpes Simplex Encephalitis in a Background of Mannose-Binding Lecithin Deficiency

**DOI:** 10.1155/2017/1429847

**Published:** 2017-02-05

**Authors:** Kenneth Asogwa, Kwame Buabeng, Amarjit Kaur

**Affiliations:** Richmond University Medical Center, 355 Bard Avenue, Staten Island, NY 10310, USA

## Abstract

Historically, psychotic disorder has been associated with viral infection. Herpes simplex infections and Epstein-Barr virus (EBV) among other viral infections have been implicated in psychotic disorder. Of note in this case report is psychotic disorder that occurred following reactivation of herpes simplex infection in a background of mannose-binding lecithin (MBL) deficiency, childhood EBV infection, and severe psychosocial stress. Herpes simplex encephalitis (HSE) remains a significant cause of morbidity and mortality despite advancement in its treatment with intravenous acyclovir. Many studies have reported psychiatric and neurological manifestation of herpes simplex infection following primary or reactivated infection, while others suggest milder clinical course of herpes simplex encephalitis in a background of immunosuppression. Another contributory factor to psychotic disorder in this case is childhood EBV exposure which has been reported to increase the risk of psychosis in adolescence and adulthood. This case report describes a 15-year-old female with MBL deficiency who presented with psychosis caused by reactivated herpes simplex infection and had good clinical recovery. Based on childhood Epstein-Barr virus exposure and psychosis in adolescence (current case), she is at increased risk of psychotic disorder in adulthood, which underscores the importance of long-term monitoring.

## 1. Introduction

Herpes encephalitis is the most common sporadic cause of encephalitis worldwide. It has an annual incidence of 2 cases per million people. It has no seasonal or gender predilection [1]. Herpes simplex encephalitis (HSE) remains a serious cause of morbidity and mortality despite advances in antiviral treatment [2]. Introduction of antiviral agent (acyclovir) reduced the mortality rate of herpes encephalitis to 6%–11% [3]; however, up to 44%–62% of survivors of herpes simplex encephalitis have neurological sequelae [4]. About a third of herpes encephalitis incidents occur in children. Herpes encephalitis in neonatal age group is largely caused by herpes simplex-2 acquired by vertical transmission during childbirth, while encephalitis after 3 months of age is usually due to herpes simplex-1. Herpes encephalitis as a cause of psychosis is rare, affecting about 1 per 200,000 cases per year. It is suggested that herpes infection like other environmental stressors causes psychosis through inhibition of N-methyl-D-aspartate (NMDA) receptor and subsequent microglia activation in the hippocampus [3]. It typically presents as psychomotor agitation, fever, headache, visual hallucination, paranoia, seizures, and altered level of consciousness [5]. It starts with prodromal symptoms which usually last for days to weeks before presenting with neurological signs and symptoms. Epstein-Barr virus (EBV) is a human herpesvirus. The majority of primary Epstein-Barr virus (EBV) infections in young children are asymptomatic. It is estimated that 90–95% of adults are seropositive to EBV. In immunocompetent hosts, it resolves and forms a harmless persistent latent infection (no active viral production). Encephalitis due to EBV is less common, usually seen in immunocompromised patients [6]. MBL is one of the collectin proteins, involved in innate immunity, and functions to activate the complement system through the lectin pathway. Deficiency of MBL defined as MBL protein that is less than 100 ng/mL is common in the general population. The clinical relevance of MBL deficiency is controversial, but it has been reported to be associated with increased susceptibility to infection if there is concomitant factor that suppresses the immunity [7].

## 2. Case Report

The patient is a biracial 15-year-old female; the father is from Sudan, and the mother is from Romania. At the time of evaluation, the patient was in high school (10th grade) and was brought to the psychiatric emergency room by an ambulance for evaluation of disorganized behavior of 24-hour duration. Upon presentation, the patient's behavior was disorganized as she paced aimlessly in the emergency room. She was restless, laughed inappropriately, murmured to herself, and was redirected verbally on multiple occasions. Her speech was disorganized, characterized by loosening of association. “I cannot control my nerves,” “I hate sounds,” and “It hurts my brain” are examples of statements she made when she presented to the emergency room. She added that she had been hearing voices but was unable to provide details. She briefly laughed, stared into the space, could not get words out, and appeared to have thought blocking. The patient also had thought broadcasting as she believed her mother could read her mind but was unable to clarify further. Collateral information from the mother revealed that the patient was under tremendous stress trying to match academic record of her elder sister who was a valedictorian. The patient had signed up for advanced courses and was under intense stress to maintain good grades in all the courses. She reported that the patient's problem started about a week prior to presentation, when it was noticed that she lacked concentration and showed increased psychomotor agitation, sudden poor academic performance, social isolation, and finally refusal to go to school. Initially, the family members thought she was having “academic stress”; hence, she was encouraged to rest for a couple of days. She was eventually brought to the hospital due to worsening symptoms: talkativeness, poor sleep, headache, paranoia, and vehement refusal to attend school. She further described that the patient became scared and suspicious of her father and classmates. She felt her classmates were talking about her; consequently, she stopped attending school. She became angry with her father, alleging that he took her cellphone charger so that she could not communicate with family members and friends. The patient denied visual and tactile hallucinations, and there was no change in her eating pattern. There was no symptom suggestive of bipolar disorder, major depressive disorder, eating disorder, anxiety disorder, head trauma, seizure, cerebrovascular accident, Parkinson's disease, or other organic brain diseases. The patient also denied past or current use of nicotine, alcohol, or other psychoactive substances; however, she used two to three cups of coffee daily to remain alert while studying. The patient denied any past psychiatric history. The past medical/surgery history included multiple episodes of streptococcal pharyngitis which started at the age of six, Epstein-Barr virus (EBV) pharyngitis which started at the age of seven, and subsequently tonsillectomy. The last episode of pharyngitis due to Group B* Streptococcus* was at the age of fourteen, which was treated with amoxicillin/clavulanate. Another significant medical history was herpes labialis at the age of fourteen which was treated with topical acyclovir. She had no known drug allergies. There is no family history of mental illness. Birth and developmental history were unremarkable. The patient denied physical, sexual, or emotional abuse and does not have any learning disability. Mental status examination revealed a medium built female with long black hair and brown eyes, who was neatly dressed. She was restless and sweaty and paced back and forth in the emergency room with poor eye contact. She was not cooperative during evaluation and had to be redirected multiple times. Speech was increased in rate, but style, volume, and rhythm were regular. The thought process showed tangentiality, circumstantiality, and blocking. Content of thought elicited was auditory hallucination and delusion of paranoid type. She denied suicidal and homicidal ideations. The patient described mood to be “worried,” while affect was congruent, inappropriate, and constricted. She was alert; however, she was disorientated to time and date. Her concentration and immediate and short-term memories were poor. The patient's impulse control was fair, but her insight and judgment were poor. Physical examination did not reveal any pathological findings. Initial laboratory tests (complete blood count, comprehensive metabolic panel, urinalysis, thyroid stimulating hormone, free thyroxin, and rapid plasma reagin) were unremarkable. Urine drug screening for the following substances was also unremarkable: oxycodone, methadone, opiates, barbiturates, phencyclidine, amphetamine, benzodiazepines, cocaine, marijuana (THC), synthetic marijuana (K2), and ethyl alcohol. Noncontrast computed tomography scan (CT scan) of the head was negative for abnormal pathology. Magnetic Resonance Imaging (MRI) could not be done because of her uncooperativeness (restlessness and inability to lie still in the scanner). The patient was admitted to the Child and Adolescent Psychiatry Unit and was treated with risperidone for psychosis and trazodone for insomnia. Her vital signs remained within the normal range during inpatient psychiatry hospitalization. On the third day of inpatient hospitalization, she remained psychotic and in addition developed lethargy, social withdrawal, photophobia, lip swelling, perioral rash, and worsening headache. Based on the new complaints, urgent consultation of a pediatrician was done, and she was transferred to the pediatric unit where emergency lumbar puncture was done. Repeat hematological test, blood chemistry, urinalysis, and pelvic ultrasound were unremarkable. Serum mannose-binding lectin was <0.5 ng/mL. Cerebrospinal fluid (CSF) analysis revealed clear cerebrospinal fluid; glucose was 67 mg/dL (normal range is 40–70 mg/dL); total protein was 21.7 mg/dL (normal range is 15–45 mg/dL), and no cell was found. CSF polymerase chain reaction (PCR) for herpes simplex-1 and herpes simplex-2 virus DNA was negative. Serological test of the CSF revealed undetectable level of N-methyl-D-aspartate (NMDA) receptor antibody and Lyme IgG/IgM antibody. Other serological tests of the CSF are reported in Table 1.

Empirical treatment with intravenous acyclovir was commenced for treatment of herpes simplex encephalitis, which was converted to Val acyclovir tablet on the third day and continued for twenty-one days. Based on DSM-V criteria, the patient was diagnosed with psychotic disorder due to another medical condition having presented with auditory hallucination, paranoid delusion, and disorganized speech (tangentiality and circumstantiality). The symptoms affected her level of functioning as she was unable to attend school. It was determined that the presenting symptoms were not due to alcohol or psychoactive drug use. Continuation of care was carried out by the psychosomatic unit, and she remained on risperidone for psychotic symptoms. She was discharged home after the presenting symptoms (psychosis, photophobia, and headache) resolved within three days of getting intravenous acyclovir. A neuropsychological test (Wechsler Adult Intelligence Scale-Revised) was done five months after discharge from the hospital, to determine any neurocognitive deficits or impairment. It must be emphasized that the patient had no baseline psychological testing for comparison. Summary of the test result indicated that the patient attained a Verbal Scale Intelligent Quotient of 99 (within average range of intelligence at the 47th percentile), a Performance Scale intelligent Quotient of 74 (within borderline range of intelligence and at the 4th percentile), and a Full Scale Intelligent Quotient IQ of 86 (categorized in the low average range of intelligence and at the 18th percentile). She is currently being followed up at the outpatient psychiatry clinic where monitoring of neurocognitive function has shown no further decline.

## 3. Discussion

MBL deficiency is associated with increased susceptibility to infection if there is a concomitant factor that suppresses the immunity [7]. Its clinical relevance in individuals without concomitant immunosuppression factors has been controversial; however, a severe form of MBL deficiency (MBL plasma levels ≤50 ng/mL) is reported to be associated with recurrent and/or severe infection especially respiratory infection [8]. Impaired immunological response due to MBL deficiency coupled with tremendous academic stress probably triggered reactivation of herpes simplex-1 infection which quickly progressed to encephalitis and psychosis. Also, the history of recurrent infection (herpes labialis infection, streptococcal pharyngitis, and EBV pharyngitis) can only be explained by the patient's severe form of MBL deficiency (level is <0.5 ng/ML) that led to immunosuppression and increased susceptibility to recurrent infection.

Viral infection as a possible cause of psychotic disorder has been described by earlier investigators. Menninger (1926) was the first to report influenza viral infection as a possible cause of schizophrenia [9]. Other infections reported to be associated with psychotic disorder include Epstein-Barr virus [10] and herpes simplex [11]. HSE typically presents as fever, headache, visual hallucination, paranoia, seizures, and altered level of consciousness [5]. HSE occurs following primary infection, or due to reactivation of latent infection in the olfactory bulb and/or in the trigeminal ganglion. Reactivation and replication of herpes simplex-1 infection in latency at the dorsal root ganglion can be triggered by stressful events [12]. Following reactivation of the virus at the dorsal root ganglion, it travels in an anterograde direction back to the peripheral skin and mucosa or to the central nervous system [13]. The exact mechanism of damage caused by herpes encephalitis is poorly understood, but it has been suggested that tissue damage caused by herpes simplex-1 infection is immune mediated [14], which explains the uncommon presentation and mild clinical course of herpes simplex encephalitis among immunosuppressed hosts [15]. Our patient presented with encephalitis and skin lesion around the oral cavity, sequelae of the anterograde migration of herpes simplex virus after reactivation. The clinical course was characterized by stable vital signs throughout hospitalization, psychosis that resolved within 3 days of intravenous antiviral treatment, and absence of neurological relapse. This mild clinical course underscores the role of immunosuppression (MBL deficiency and psychosocial stress) in pathogenesis of herpes simplex encephalitis.

It is suggested that HSV psychosis is caused by inhibition of NMDA receptor. The inhibition is caused by a cascade of events initiated by HSV glycoprotein on microglia/astroglia. The NMDA inhibition can also be through direct inhibition by HSV antibody [3]. Therefore, HSV psychosis is still plausible even in the absence of HSV antibody; in such situation, NMDA receptor becomes inhibited through the alternate route initiated by HSV glycoprotein on microglia/astroglia.

Diagnosis of HSE in immunosuppressed patients has always been challenging. Even though CSF antibodies to herpes simplex virus are not useful in making the diagnosis of HSE, a report by Kurtz (1974) showed a significantly elevated level of immunoglobulin-G in a HSE patient with prior history of cold sore which has similar presentation to the patient described in this report [16]. Choice of diagnosing herpes simplex encephalitis is through detection of HSV-1 DNA using PCR technique; however, multiple studies have shown PCR studies with false negative result usually in the early course of HSE [17–19].

Radiological evidence of herpes encephalitis is cerebral edema and contrast enhancement. Magnetic Resonance Imaging (MRI) remains the most sensitive and specific radiological test to diagnose herpes encephalitis. Computerized Tomography (CT) scan of the brain is positive in about 50% of cases and usually negative during the initial 4–6 days of the illness. In this patient, CT scan of the head was negative; however, it was done within 4 days of onset of illness. And more sensitive and specific radiological test (MRI of the brain) was not done due to patient uncooperativeness.

Neurocognitive decline has been documented extensively as one of the sequelae following HSE, hence the need to monitor neuropsychological function following HSE. According to Hokkanen et al. (1996), there is a decline in neuropsychological function (memory, visuoperceptual, or verbal functions) following HSE [20]; this finding is similar to the decline in Performance Scale Intelligent Quotient in our patient. It is important to state that there was no baseline psychological test for comparison with WAIS-R which was done 5 months after hospitalization. The patient's age at the time of the psychological testing was 16 years which explains the choice of Wechsler Adult Intelligence Scale-Revised (WAIS-R) over Wechsler Intelligence Scale for Children (WISC).

Recommended treatment of choice of HSE is intravenous acyclovir [4]; however, the outcomes of treatment with oral valacyclovir have also been evaluated and it may be an acceptable treatment option in resource limited settings [21]. In this case, the choice of the antiviral agent for the herpes simplex infection administered empirically was intravenous acyclovir for three days. Subsequently, valacyclovir was started and was continued for twenty-one days with satisfactory clinical response. Risperidone was started immediately at the psychiatry emergency room to address psychosis but was discontinued upon discharge from the hospital.

This patient presented with chronic EBV infection; it is imperative to review how previous EBV infection contributes to current presentation of psychosis. There is an association between serological evidence to EBV infection and subclinical psychotic experience [10]. The psychotic disorder in this case report was due to HSE; however, she was already at risk of psychosis based on a prospective study which showed that childhood EBV exposure is associated with psychotic disorder in adolescence [22]. She continues to be at increased risk of psychotic disorder in adulthood considering findings of some epidemiological studies [22, 23]. This highlights the role of infection and immunological dysfunction in this case presentation and emphasizes the importance of long-term follow-up as she continues to be at risk of psychotic disorder in adulthood.

Studies have shown reduction in mortality rate following treatment of HSE with acyclovir; however, survivors had moderate to severe impairment between 31 and 54% [4, 24]. The impairment was caused by cognitive decline, seizure disorder, hemiparesis, and speech impediment. Factors like age (<30 years old), level of consciousness at onset of therapy (Glasgow Coma Scale >10), and disease duration before the onset of therapy (initiation of treatment within 4 days) lead to good outcome [25]. In this case, age below 30 years and prompt initiation of intravenous acyclovir/oral valacyclovir may explain the mild neuropsychological impairment and absence of neurological relapse to date. Clinical monitoring is imperative as she remains at risk of psychosis in adulthood and also to observe the course of the neuropsychological impairment (decline in visuoperceptual function).

## Figures and Tables

**Table 1:** 

Epstein-Barr virus monospot	Negative

Epstein-Barr virus (EBV) capsid antigen IgM antibody	<0.91 (negative: <0.91; equivocal: 0.91–1.09; positive: ≥1.10)

Epstein-Barr virus (EBV) capsid antigen IgG antibody	2.20 (negative: <0.91; equivocal: 0.91–1.09; positive: ≥1.10)

Epstein-Barr virus (EBV), Epstein-Barr virus nuclear antigen (EBNA) IgG antibody	5.00 (negative: <0.91; equivocal: 0.91–1.09; positive: ≥1.10)

Herpes simplex-1 IgG antibody	5.00 (negative: <0.91; equivocal: 0.91–1.09; positive: ≥1.10)

Antideoxyribonuclease-B (anti-DNase-B)	<95 U/mL (normal range: <376 U/mL)

Varicella-Zoster antibody IgM	<0.90 (negative: <0.91; equivocal: 0.91–1.09; positive: ≥1.10)
